# Cloning and Expression of Pigeon-Derived *Escherichia coli* Type 1 Pilus Clusters and Analysis of Amino Acid Sequence Characteristics of Functional Proteins

**DOI:** 10.3390/genes15101253

**Published:** 2024-09-26

**Authors:** Junhong Chen, Wei Dai, Hang Wang, Weiqiang Lei, Guangyuan Fang, Dingzhen Dai

**Affiliations:** 1 School of Animal Science and Food Engineering, Jinling Institute of Technology, Nanjing 210046, China; chenjunhong@jit.edu.cn (J.C.); daiwei@jit.edu.cn (W.D.); leiweiqiang@jit.edu.cn (W.L.); fgy@jit.edu.cn (G.F.); 2 College of Veterinary Medicine, Yangzhou University, Yangzhou 225009, China; wh3072@gmail.com

**Keywords:** pigeon-derived *Escherichia coli*, type 1 pili recombinant bacteria, D-mannose sensitive hemagglutination, amino acid sequence

## Abstract

Background: Type 1 pili, as an important virulence factor of *E. coli*, has certain homology between APEC and UPEC, but the homology degree is not clear enough. Objectives: This study aims to compare the homology between them. Methods: The recombinant bacteria were constructed by homologous recombination. The pili were observed by TEM, and the hemagglutination characteristics were determined by MHSA. The complete gene sequence was determined by sequencing, and the amino acid sequences of the functional proteins of type 1 pili of APEC and UPEC were compared. Results: TEM showed that they could express pili, which were slender, straight, and dense. Stable-pUC-*fimBH* has MHSA but stable-pUC-*fimBG* does not. The amino acid sequence similarity of FimB of NJ05 and UPEC was 98.8%, FimE was 99.4%, and the similarity between them was 51.5%. Compared with UPEC’s type 1 pili FimC and FimD sequences, the similarity was 99.52% and 87.8%, respectively. The amino acid sequence of FimA of NJ05 was 89–96%, similar to UPEC, and the N-terminal and C-terminal amino acid sequences were exactly the same. The gene sequence and amino acid sequence similarity of FimH between them were both above 99%. The similarity of the pilus binding domain of FimH was 52.8%, but only 27.6% in the receptor binding domain. A few of the same amino acid residues were found in the corresponding regions of FimA, FimF, FimG, and FimH. Conclusions: The type 1 pili of APEC and UPEC come from the same origin, which is helpful to further reveal the pathogenic mechanism of *E. coli* infection in the poultry respiratory tract.

## 1. Introduction

**Figure 1:**
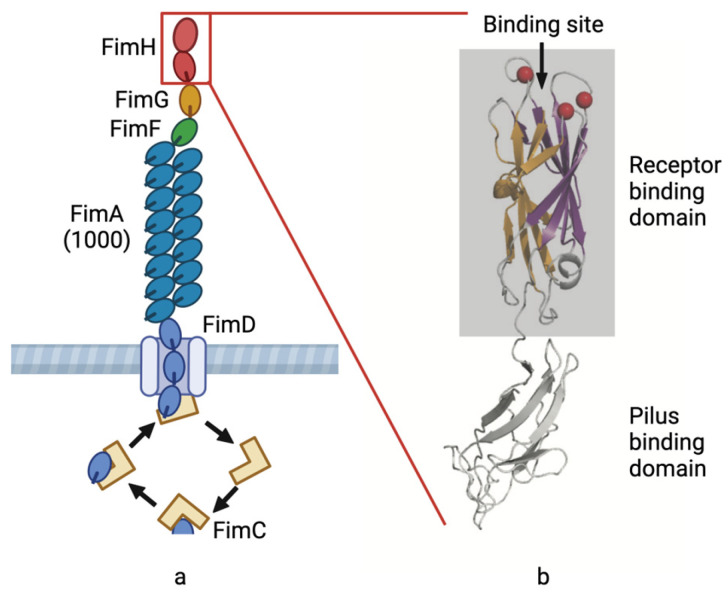
APEC type 1 pilus structural pattern. (**a**) Type 1 pili are composed of a pilus rod and a pilus tip. The pilus rod is composed of numerous FimA protein subunits, and the pilus tip is connected to the pilus rod by the adhesin FimH protein through the FimF and FimG proteins. (**b**) The FimH protein is composed of a receptor binding domain and a pilus binding domain. The connection between FimF and FimG proteins is completed by the pilus binding domain, while the adhesion to the mannose receptor is achieved by the receptor binding domain. The binding site is indicated by arrows.

Avian colibacillosis is caused by avian pathogenic *Escherichia coli* (APEC), which often causes balloon inflammation, pericarditis, perihepatic inflammation, omphalitis, yolk peritonitis, and even septic death. APEC infects birds through the respiratory tract. Depending on the adhesion of bacterial type 1 pili to mannose receptors in respiratory epithelial cells, bacteria can settle, reproduce, and spread throughout the body [1,2]. *E. coli* is the main bacterial pathogen that infects pigeons. Indeed, *E. coli* is usually isolated from pigeons with adenovirus or herpesvirus infections. Therefore, it might be considered to be a secondary pathogen. Some APEC also have type P pili, which can provide adhesion for further infection [3]. Type 1 pili are protein protuberant, which is also an important virulence factor of human uropathogenic *Escherichia coli* (UPEC). It can adhere to the mannose receptor of human urethral epithelial cells and cause urethral infection [4,5,6]. The hemagglutination property of type 1 pili can be blocked by D-mannose and has D-mannose sensitive hemagglutination (MSHA) properties. Some antagonists can block the binding of type 1 pili to the mannose receptor, based on which drugs can be screened to prevent the invasion of *E. coli* [7]. Type 1 pili, which is controlled by a gene cluster of approximately 9 kb, is located on bacterial chromosomes and consists of *fimB*, *fimE*, *fimA*, *fimI*, *fimC*, *fimD*, *fimF*, *fimG*, and *fimH* genes [8,9]. Among them, *fimB*, *fimE*, and *fimI* are regulatory genes that control the expression of pili [10]. The chaperone protein FimC encoded by the *fimC* gene is responsible for the transport of pili subunits [11]. The propulsion protein FimD encoded by *fimD* assembles the transported subunits into protuberant pili [12]. *fimA*, *fimF*, *fimG*, and *fimH* are structural genes, and the pili is formed by winding many FimA subunits encoded by *fimA*. The top protein FimH with adhesion recognition function is encoded by *fimH*. The proteins encoded by *fimF* and *fimG* are used to connect the pilose stem and the top protein. The four components constitute the complete structural components of type 1 pili. FimH has two domains: the pilus binding domain and the receptor binding domain (Figure 1) [13,14,15].

Studies have shown that APEC also has type 1 pili. It has been reported that APEC has some homology with UPEC type 1 pilis *fimA* and *fimH*, but for the whole type 1 pilus gene cluster, the complete amino acid sequence of APEC pili and its correlation with other types of pili protein sequences are not clear. Pili is not only an adhesion factor, but also a good immunogen [16,17]. In recent years, it has been found that FimH can activate the TLR4 receptor, induce the secretion of inflammatory factors [18,19], and act as an immune adjuvant for tumor immune prevention [20]. The results showed that there was a variation in the amino acid composition of UPEC type 1 pili [21], and the homology of amino acids with APEC type 1 pili was not clear. In addition, as a variant type of type 1 pili, type 1C pili has certain homology with part of the gene sequence of type 1 pili, although it is completely different in hemagglutination and adhesion [22,23]; however, the degree of homology is not clear enough. For the prevention and treatment of avian colibacillosis, the study of the expression of APEC type 1 pili gene clusters and the analysis of amino acid sequence characteristics are not only helpful in revealing the pathogenic mechanism, heredity, and variation of APEC but are also of great significance for screening pilus antigens to prepare vaccines to prevent avian colibacillosis.

In view of the homology of some genes in the type 1 pilus gene cluster of APEC and UPEC, primers were designed in the regions with the same upstream and downstream gene sequences of the hypothetical type 1 pili gene cluster. The whole gene of type 1 pili of *E. coli* isolated from pigeon was amplified, cloned, and expressed, and the whole gene sequence of type 1 pili was determined. The amino acid sequences of each functional protein were compared by software, and the correlation with the same functional protein sequence of other types of pili was analyzed, which provided support for revealing the adhesion mechanism of pili and screening the type 1 pilus antigen.

## 2. Materials and Methods

### 2.1. Strains

APEC NJ05 was preserved by the Laboratory of Veterinary Microbiology, School of Animal Science and Food Engineering, Jinling Institute of Technology. Electron microscopy and D-mannose sensitive hemagglutination assay (MSHA) confirmed that NJ05 has type 1 pili. A host bacteria, stable, chemically competent cell for the transformation of recombinant plasmids was purchased from Shanghai Weidi Biotechnology Company, Shanghai, China.

### 2.2. Primer Design

Based on the sequences of the type 1 pili gene (GenBank: CP005930.1), Primer 5.0 software was used to design *fimBH* and *fimBG* primers. Primer *fimBH* is to amplify the gene cluster of type 1 pili from *fimB* to *fimH*; the expected size of the amplified gene fragment is approximately 8924 bp. Primer *fimBG* is to amplify the gene cluster of type 1 pili from *fimB* to *fimG*; the expected size of the amplified gene fragment is approximately 7927 bp. Compared with the complete sequence of the type 1 pili gene cluster, only the *fimH* gene encoding adhesion protein was deleted. The primer sequences are shown in Table 1. Hind III and EcoR I digestion sites (underlined) were introduced into the primers. Part of the gene in the polyclonal site of the pUC19 plasmid (blackbody) was added at the end of the primer.

### 2.3. DNA Extraction

APEC NJ05 was cultured for 16–24 h, and bacteria were obtained by centrifugation. Total DNA was isolated using a DNA kit (TIANGEN DP302, Beijing, China) according to the instructions.

### 2.4. PCR Amplification, Cloning, and DNA Purification

The reaction condition was pre-denatured at 98 °C for 5 min, 35 cycles of 95 °C for 30 s, 60 °C for 10 s, and 72 °C for 5 min, then extended at 72 °C for 10 min and maintained at 16 °C for 5 min. PCR products were analyzed by 1.0% agarose gel electrophoresis. DNA purification was performed using the Gel & PCR Clean Up Kit (Omega Bio-tek, Inc., D2000, Norcross, GA, USA).

### 2.5. Construction of the Recombinant Bacteria

The amplified products obtained by PCR amplification were subjected to restriction digestion, homologous combination ligation, and PCR screening to obtain the type 1 pili gene cluster recombinant plasmids pUC-*fimBH* and pUC-*fimBG*. The recombinant plasmids were transformed into the host bacteria stable, and the positive clones were screened by ampicillin resistance. The full sequence of the type 1 pili gene cluster was determined by extracting the plasmids, confirming that the recombinant bacteria Stable-pUC-*fimBH* and stable-pUC-*fimBG* expressing the APEC type 1 pili gene cluster recombinant plasmid were obtained.

### 2.6. Transmission Electron Microscopy (TEM)

APEC NJ05, stable-pUC-*fimBH*, stable-pUC-*fimBG*, and stable host bacteria were cultured at 37 °C for 16–24 h with shaking at 250 rpm. A small amount of bacterial droplets was absorbed on the glass plate; the copper mesh was placed on the bacterial liquid facing down. After approximately 10 min, the copper mesh was removed, followed by negative staining of 5 min with 2% phosphotungstic acid, drying under an incandescent lamp, observation under an electron microscope, and photo preservation.

### 2.7. Hemagglutination Assay and D-Mannose Sensitive Hemagglutination Assay (MSHA)

Wild-type strains NJ05 and recombinant bacteria Stable-pUC-*fimBH* were inoculated into 5 mL LB (Amp+) medium at 37 °C and cultured with 230 rpm for 8–10 h. An amount of 50 μL of bacteria solution was mixed with the same amount of 1% pigeon-derived red blood cells and gently oscillated; slide agglutination reaction was then carried out, and hemagglutination was observed. Agglutination was determined to be positive.

D-mannose sensitive hemagglutination of wild-type NJ05 bacteria was determined. An amount of 50 μL of hemagglutination-positive NJ05 bacterial solution was mixed with an equal volume of 5% D-mannose. This was incubated at room temperature for 10 min, then 50 μL of red blood cells was added and mixed well. The slide was gently shaken for 3–10 min and the results observed to determine whether or not mannose can inhibit hemagglutination.

### 2.8. Complete Gene Sequence Determination

The complete gene sequence of the type 1 pili gene cluster was determined. The nucleic acid sequence was determined by sending the universal sequencing primers M13 rev and M13 fwd to the GenScript company (Nanjing, China) to undergo next-generation sequencing (NGS). The gene sequence was analyzed using DNAstar version 17.6 analysis software and translated into structural gene proteins.

### 2.9. Amino Acid Sequence Analysis

The amino acid sequence of the functional protein of the type 1 pili gene cluster was analyzed. SnapGene version 4.3 and online software Clustal version 2.0 were used to analyze the gene sequences and amino acid sequences of the pili FimA, FimB, FimE, FimC, FimD, and FimH proteins from different sources, and these were compared with the amino acid sequences of related proteins such as type P pili PapC, PapD of the APEC 83972 strain (GenBank: DQ010312.1), and type 1C pili FocA and FocH of the UPEC ABU 83972 strain (GenBank: AM690762.1) to analyze the homology and variation.

## 3. Results

### 3.1. Construction of Recombinant Bacteria and Expression of Pili

#### 3.1.1. Results of PCR Amplification of *fimBH*

**Figure 2:**
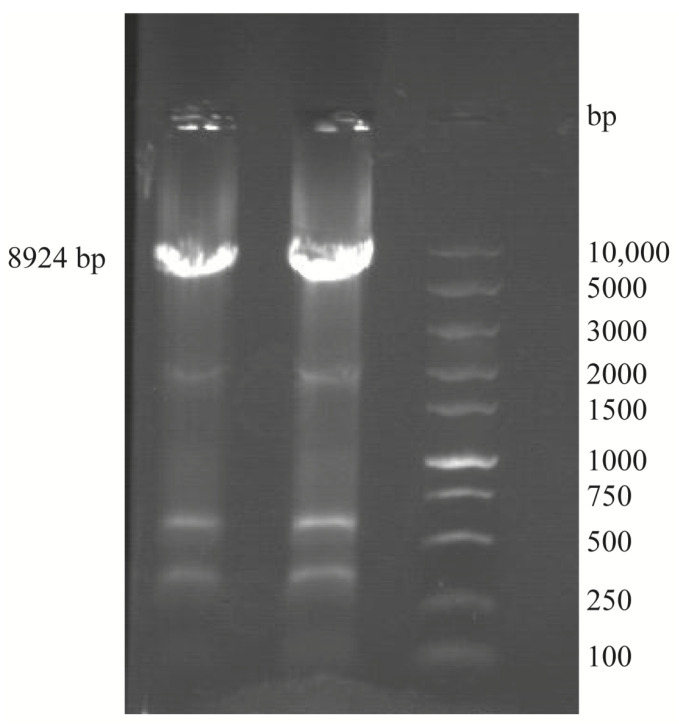
The agarose gel electrophoresis results of PCR amplification products of *fimBH* using primer 1. Abbreviations: 1-2, *fimBH*; M, 10,000 bp DNA Maker.

Electrophoresis analysis showed that a DNA fragment of approximately 10,000 bp was amplified from the chromosome of pigeon-derived *E. coli* NJ05 using primer 1, which was close to the estimated 8924 bp (Figure 2).

#### 3.1.2. Results of PCR Amplification of *fimBG*

**Figure 3:**
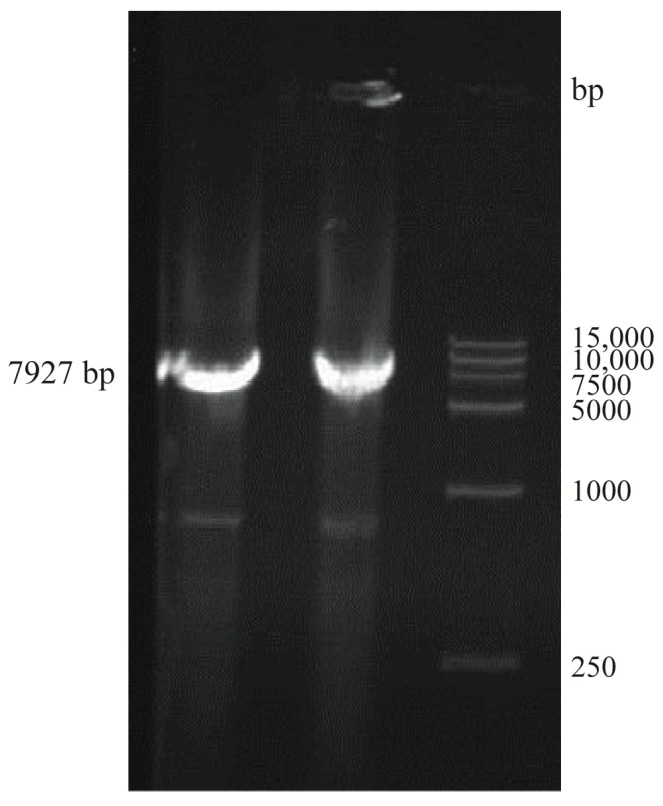
The agarose gel electrophoresis results of PCR amplification products of *fimBG* using primer 2. Abbreviations: 1-2, *fimBG*; M, 15,000 bp DNA Maker.

Electrophoresis analysis showed that a DNA fragment with a size between 7500 and 10,000 bp was amplified from the chromosome of pigeon-derived *E. coli* NJ05 using primer 2, which was close to the estimated 7927 bp (Figure 3).

#### 3.1.3. Hemagglutination and MSHA

**Figure 4:**
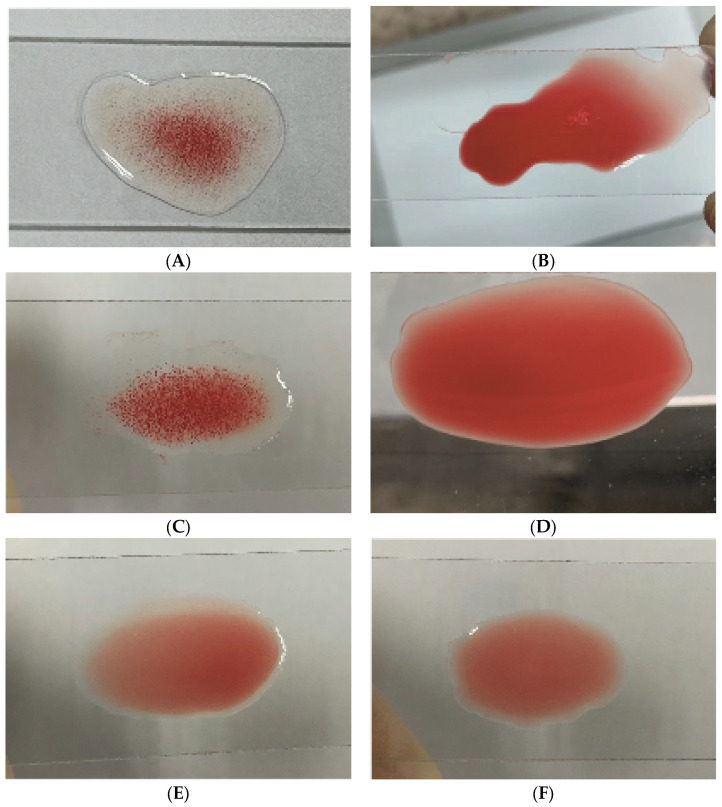
The hemagglutination of NJ05 (**A**), stable-pUC-*fimBH* (**C**), stable-pUC-*fimBG* (**E**), and stable (**F**). D-mannose sensitive hemagglutination of NJ05 (**B**) and stable-pUC-*fimBH* (**D**).

The wild *E. coli* NJ05 strain has the property of agglutinating red blood cells (Figure 4A), and the application of D-mannose can block the hemagglutination of NJ05 (Figure 4B). The recombinant bacteria stable-pUC-*fimBH* can also agglutinate red blood cells (Figure 4C), can be blocked by mannose (Figure 4D), and have the typical MSHA characteristics of type 1 pili. The recombinant bacteria stable-pUC-*fimBG* do not have hemagglutination properties (Figure 4E), so there is no MSHA. The host bacteria stable did not express pili and therefore do not have hemagglutination properties (Figure 4F).

#### 3.1.4. Determination of the Complete Gene Sequence of Type 1 Pili

**Figure 5:**
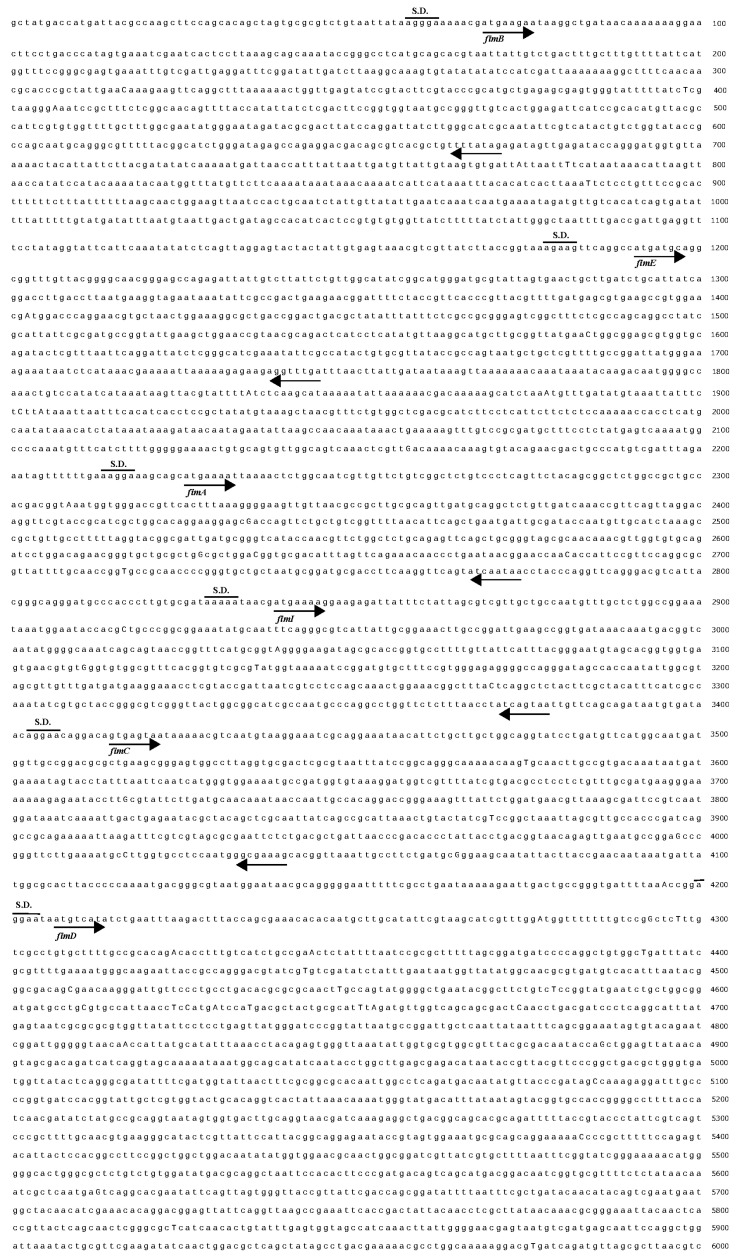

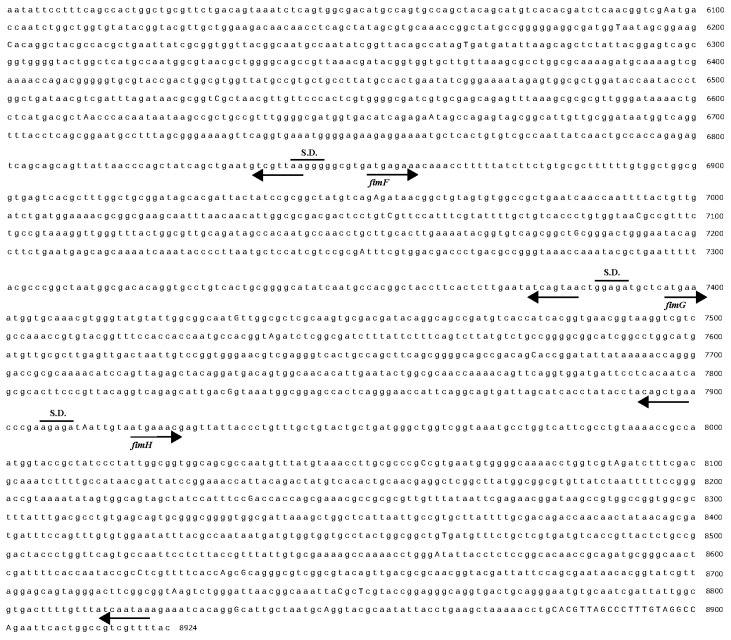
Complete gene sequencing of type 1 pili gene cluster of *E. coli* from pigeon. Ribosomal binding sites and potential start and stop points of the genes are indicated with the arrows, “→”, start, and “←”, stop.

After electrophoresis identification, the nucleic acid sequence was determined by sending the DNA to a biotechnology company using universal sequencing primers M13 rev and M13 fwd. The gene sequence was analyzed using SnapGene analysis software and translated into the amino acid sequence of the protein. It was found that the gene sequence and amino acid sequence were consistent with type 1 pili and the reading frame was correct. The determined size of the *fimBH* gene cluster was 8753 bp (calculated from the start codon of *fimB*), which contained *fimB*, *fimE*, *fimA*, *fimI*, *fimC*, *fimD*, *fimF*, *fimG*, and *fimH* gene analysis by SnapGene software (Figure 5). The size of the *fimBG* gene was 7831 bp (calculated from the start codon of *fimB*), which contained *fimB*, *fimE*, *fimA*, *fimI*, *fimC*, *fimD*, *fimF*, and *fimG* genes.

#### 3.1.5. TEM Observation of Type 1 Pili

**Figure 6:**
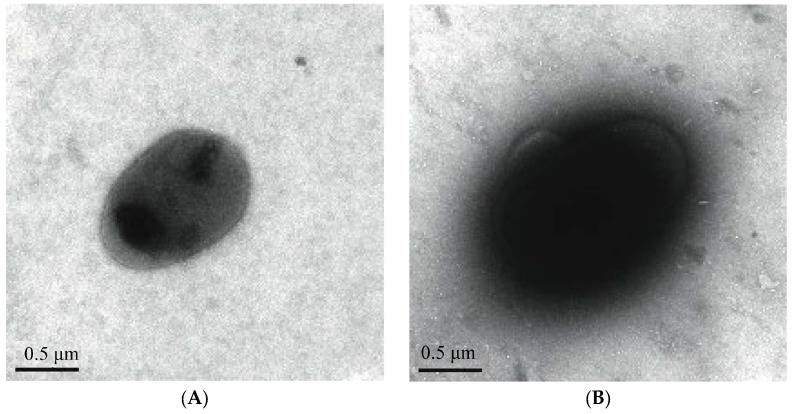

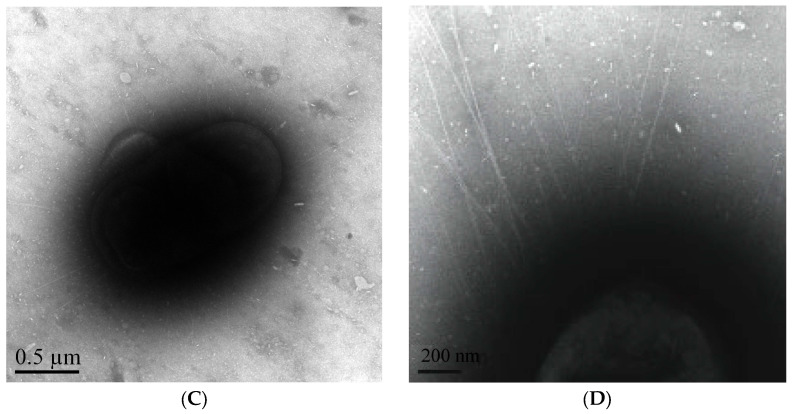
Observation of host bacteria stable, wild bacteria, and recombinant bacteria under TEM. (**A**) Host bacteria stable without pili. (**B**) Wild bacteria NJ05 with pili. (**C**) The recombinant bacteria stable-pUC-*fimBH* with pili. (**D**) The recombinant bacteria stable-pUC-*fimBG* with pili.

The host bacteria stable did not have pili (Figure 6A), and the wild *E. coli* NJ05 expressed pili (Figure 6B). The recombinant bacteria stable-pUC-*fimBH* has pili, which are slender, straight, and dense in number (Figure 6C). Although the recombinant strain stable-pUC-*fimBG* lacking the *fimH* gene could not agglutinate red blood cells, it was still able to express pili (Figure 6D), and its morphology was not significantly different from that of the recombinant strain stable-pUC-*fimBH* and the wild strain NJ05.

### 3.2. Comparison of Amino Acids of Pilus Rod FimA Proteins from Different Sources

**Figure 7:**
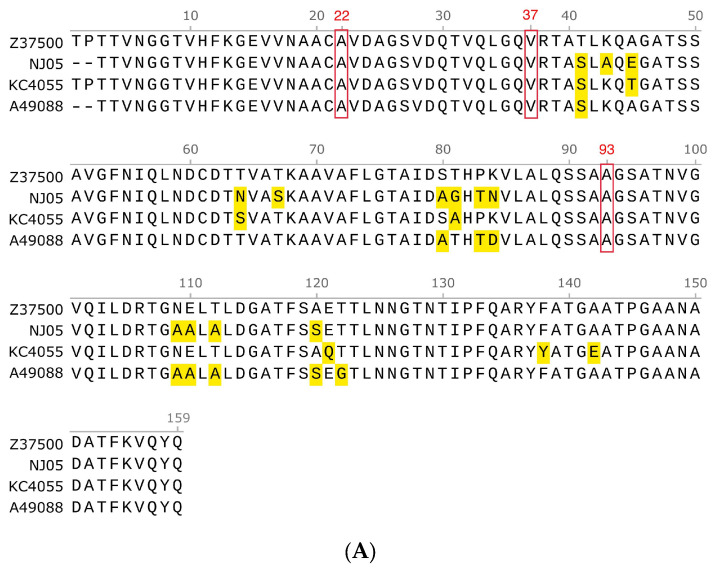

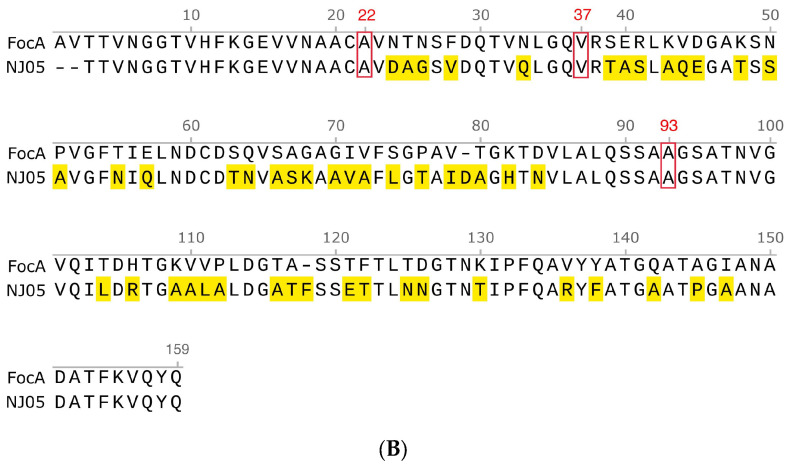
Comparison of amino acids of pilus rod FimA proteins from different sources. (**A**) The amino acid sequence alignment of the FimA proteins of Z37500, NJ05, KC4055, and A49088 was performed using MUSCLE in SnapGene software. (**B**) The amino acid sequence alignment of FimA subunits of NJ05 and FocA subunits of F1C fimbriae. Amino acids that differ from the first line amino acid sequence are highlighted in yellow. The identical alanine A at position 22, valine V at position 37, and alanine A at position 93 are shown with red boxes.

The FimA protein subunit has 182 amino acids, and the signal sequence is MILSVIAGAVAMAVVSFGVNA. In the compared sequences, the amino acid sequences of the FimA subunit of type 1 pili of the NJ05 strain and other APEC strains were 89 to 96% similar. The homologous regions were mainly located at the N-terminus, middle, and C-terminus, and the amino acid sequences of the homologous regions were exactly the same. Based on the sequence position of Z375001, the variable regions were mainly located between 41–45, 80–84, 109–112, and 120–123 (Figure 7A).

The FimA of strain NJ05 of APEC has only 67% homology with the amino acid sequence of the FocA (GenBank: AM690762.1) of strain ABU 83972 of UPEC. However, the amino acids A22, V37, and A93, used to connect the pilus rod subunits, have not changed; both positions 8 and 14 are glycine G (Figure 7B).

### 3.3. Comparative Analysis of Amino Acid Sequences of Regulatory Proteins fimB and fimE

**Figure 8:**
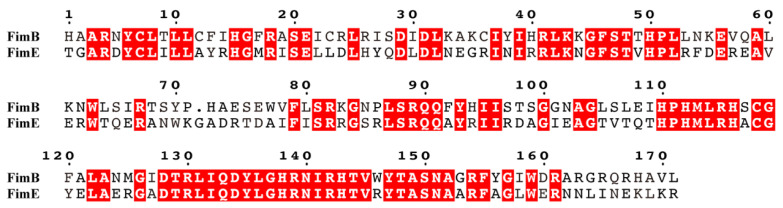
Comparison of the amino acid sequences of the FimB and FimE proteins of NJ05 strain type 1 pili. The identical amino acids are highlighted in red.

By comparison, it was found that the amino acid sequence similarities of the mature FimB and FimE proteins of type 1 pili of NJ05 and UPEC were 98.8% and 99.4%, respectively, with only a few amino acids mutated. There was a high degree of homology between FimB and FimE, with 88 identical amino acids at corresponding positions in the mature proteins, accounting for 51.5% of the amino acids in the mature proteins. In particular, a sequence consisting of 25 amino acids (^128^DTRLIQDYLGHRNIRHTVWYTASNA^152^) differed only at position 146, with W for FimB and R for FimE, and the similarity was as high as 96% (Figure 8).

### 3.4. Comparative Amino Acid Sequence Analysis of FimC Chaperone Proteins

The amino acid sequences were essentially identical, with 99.52% similarity compared to human UPEC type 1 pili FimC, differing in only one amino acid. The SD sequence is AGGAA and the signal peptide consists of 36 amino acids and has a start codon GTG, encoding valine, which is different from methionine as the start codon. Since type P pili and type 1 pili tend to coexist in UPEC and APEC [24,25], they were also used as the target of comparison in some of the sequence comparisons. FimC and PapD proteins are also chaperone proteins. The amino acid homology between type 1 pili FimC of strain NJ05 and type P pili PapD of strain 83972 (GenBank: DQ010312.1) was only 32.9%, but there were many homology patches between the two proteins.

**Figure 9:**
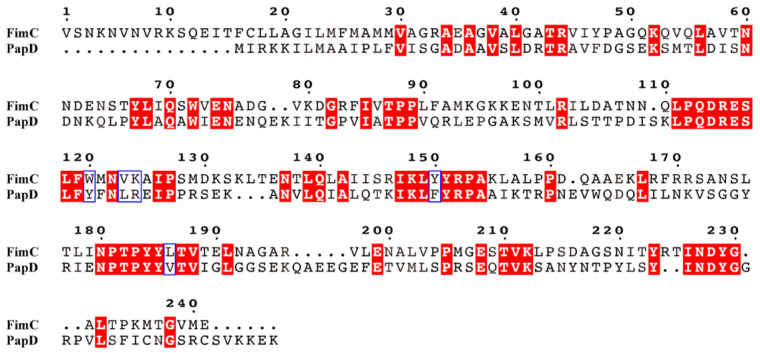
Comparison of amino acid sequences of type 1 pili FimC in NJ05 strain and type P pili PapD in strain 83972 (GenBank: DQ010312.1). The identical amino acids are highlighted in red. The amino acids that differ in the blue boxes are similar residues.

In the sequence consisting of a total of 213 amino acids, there were as many as 7 consecutive identical amino acids of more than 3 amino acids, among which ^111^LPQDRESLF^119^ was the largest homology patch. There were differences in F and Y between ^147^IKLYYRPA^154^ of FimC and IKLFYRPA of PapD, but F and Y were functionally identical amino acids. Similarly, the same situation exists between ^180^NPTPYYLTV^188^ and NPTPYYVTV, where only the L and V amino acids differ, and L and V also have the same functions. In particular, the 17 amino acids, ^111^LPQDRESLFWMNVKAIP^127^, in the middle of the protein were connected together, 9 consecutive amino acids were identical, and the 5 different ones were mostly amino acids with the same function (Figure 9).

### 3.5. Amino Acid Sequence Analysis of FimD Protein

The signal peptide of FimD protein has 37 amino acids, and the mature protein has 841 amino acids. Compared with the sequence of type 1 pili FimD of human UPEC, there were 87.8% identical amino acids in the corresponding positions.

**Figure 10:**
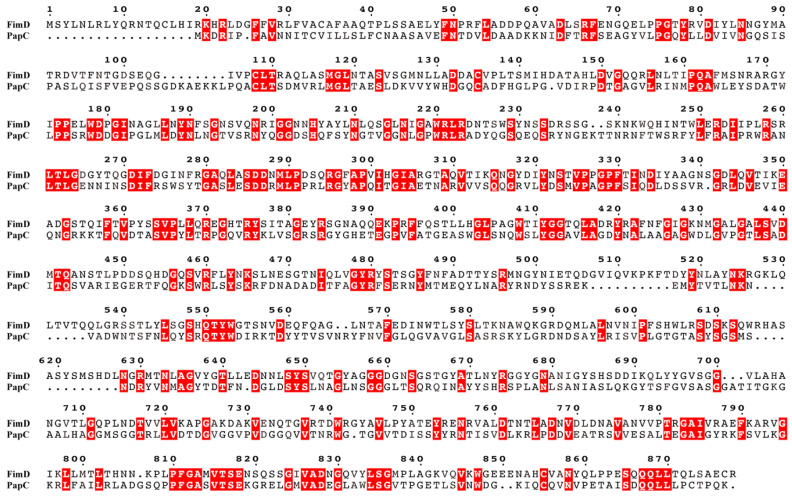
Comparison of amino acid sequences of type 1 pili FimD in NJ05 strain and type P pili PapC in strain 83972 (GenBank: DQ010312.1). The identical amino acids are highlighted in red.

Compared with the PapC pilus propulsion protein sequence of type P pili, the protein size was similar, and there was a large number of homology patches between the two, among which there were 15 places with the same arrangement of three consecutive amino acids, namely ^108^CLT^110^, ^117^MGL^119^, ^161^PQA^163^, ^271^DIF^273^, ^285^SDD^287^, ^289^MLP^291^, ^303^GIA^305^, ^328^GPF^330^, ^364^SVP^366^, ^411^YGG^413^, ^478^GYR^480^, ^648^SYS^650^, ^783^GAI^785^, ^826^VAD^828^, and ^834^LSG^836^. There were four consecutive amino acids in the same order at 6 locations: ^220^WRLR^223^, ^261^LTLG^264^, ^550^QTYW^553^, ^809^PFGA^812^, ^815^VTSE^818^, and ^867^QQLL^870^, respectively. The results showed that there was a certain degree of homology in the amino acid sequences between the propulsion proteins in the assembly process of different types of pili (Figure 10).

### 3.6. Amino Acid Sequence Analysis of FimH Protein

Compared with the type 1 pili FimH of UPEC, the gene sequence and amino acid sequence similarity of the type 1 pili FimH protein of NJ05 were both above 99%. The amino acid sequence of the type 1 pili FimH protein was very different from that of the type P pili papG protein. In fact, the amino acid sequence of the papG protein itself was very different. Twelve genotypes have been found, and the papG gene of APEC found so far mostly belongs to genotype 11 [26].

### 3.7. Comparison of Amino Acid Sequences of Receptor Binding Domains of FimH and FocH Proteins

**Figure 11:**
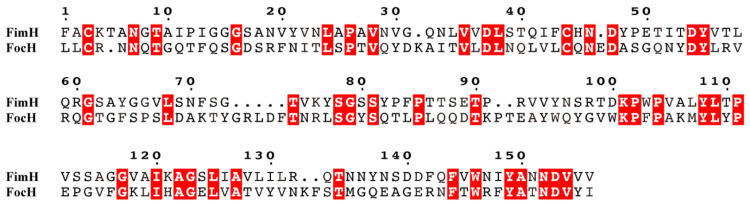
Comparison of amino acid sequences of receptor binding domains of type 1 pili FimH of the NJ05 strain and type 1C pili FocH of the ABU 83972 strain (GenBank: AM690762.1). The identical amino acids are highlighted in red.

The results of the amino acid sequence alignment of the FimH protein and the receptor binding domain of the type 1C pili FocH protein are shown in Figure 11. In the aligned sequence consisting of 156 amino acids, only 43 amino acids were identical, and the identical amino acid residues accounted for only 27.6%. In particular, the key amino acid mutations in the “binding bag” related to mannose receptor adhesion were the most obvious. The “binding bag”-related amino acid sites include F1, N46, D47, D54, Q133, N135, N138, D140, D141, and F142, which are mostly hydrophilic amino acids. The comparison results revealed that the amino acids F1, Q133, N135, N138, D140, D141, and F142 of the “binding bag” were mutated. The three amino acids Y48, I52, and Y137 of the “bag mouth” that make up the “tyrosine gate” were completely different, which was also the main reason why type 1C pili no longer bind to mannose receptors.

### 3.8. Comparison of Amino Acid Sequences of the Pilus Binding Domains of FimH and FocH Proteins

**Figure 12:**
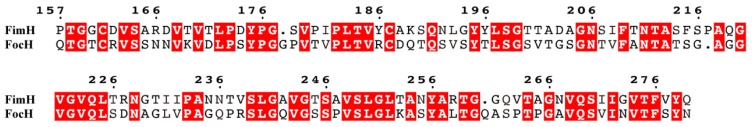
Comparison of amino acid sequences of the pilus binding domains of type 1 pili FimH of the NJ05 strain and type 1C pili FocH of the ABU 83972 strain (GenBank: AM690762.1). The identical amino acids are highlighted in red.

The amino acid sequence alignment of the pilus binding domains of the FimH protein and the FocH protein is shown in Figure 12. The four amino acids at positions 157–160 form a connecting loop of PTGG, and the connecting loop of FocH was composed of QTGT, half of which were mutated. The amino acid sequence of the pilus binding domain had a total of 123 amino acids, and the amino acid residues corresponding to 65 positions were identical, reaching 52.8%, and there were many homology patches at the corresponding positions. The largest patches were ^220^GVGVQL^225^.

### 3.9. Comparison of Amino Acid Sequences of FimH, FimA, FimF, and FimG Proteins

**Figure 13:**
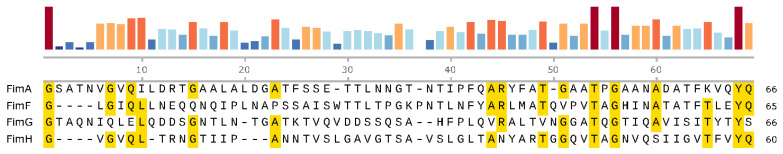
Comparison of partial amino acid sequences of FimH, FimA, FimF, and FimG proteins. The identical amino acids are highlighted in yellow. The sequence conservation is shown as colored bars.

The analysis also revealed that the amino acid residues of the FimA, FimF, FimG, and FimH proteins had very low similarity with each other, but some identical amino acid residues can still be seen in some corresponding positions (Figure 13), especially G1, T54, G56, and Y68, which were all the same for the four proteins.

## 4. Discussion

The complete type 1 pili gene cluster is composed of *fimB*, *fimE*, *fimA*, *fimI*, *fimC*, *fimD*, *fimF*, *fimG*, and *fimH* genes, which have the biological function of independently expressing pili and are located on the chromosomes of bacteria. They encode FimB, FimE, FimA, FimC, FimD, FimF, FimG, and FimH proteins, respectively. The main structure of type 1 pili is the pilus rod. The FimA protein subunits encoded by *fimA* are formed through mechanical combination, and more than 1000 FimA protein subunits are entangled to form the pilus rod [8,9]. FimH is an adhesion protein that recognizes the mannose receptor, and FimF and FimG are used to connect the pilus rod and adhesion protein. To study the structure and function of pili, it is necessary to construct a type 1 pilus expression vector and test the formation of pili [11]. The expression function of type 1 pili depends on whether or not the functional proteins of type 1 pili are present, and the strength of the function of each functional protein depends on the primary structure of the protein, that is, the amino acid sequence. Currently, there are many studies on the expression of type 1 pili and the structural function of pilus proteins in UPEC, but there are few studies on the expression and structural composition of type 1 pili in APEC. In this study, homologous recombination was used to construct the type 1 pilus gene cluster expression plasmid vector and the *fimH* deletion expression vector. The results showed that the APEC type 1 pilus plasmid expression vector can express complete pili without losing the hemagglutination property, and the plasmid lacking the *fimH* gene can also independently express pili, which have no significant morphological differences from complete type 1 pili, but they have lost the mannose-sensitive hemagglutination property (MSHA), which also affects the ability of the pili to adhere to host epithelial cells.

Sequence analysis showed that the type 1 pilus gene cluster of pigeon-derived *E. coli* NJ05 had complete *fimB*, *fimE*, *fimA*, *fimC*, *fimD*, and *fimH* genes. The amino acid sequences of FimB, FimE, FimA, FimC, FimD, and FimH proteins were inferred based on the gene sequences, and the homology of the amino acid sequences was compared and analyzed. Studies have shown that, compared with the type 1 pili of UPEC, the regulatory protein sequences of FimB, FimE, FimC, and FimD of the type 1 pili of the NJ05 strain of APEC are highly homologous. However, FimA, as a structural protein, has significant variation in its encoding gene and large differences in its amino acid sequence [21], but no one has classified it yet. FimH, an adhesion protein, has less variation [27]; in particular, the *fimH* pilus binding domain gene sequence located at the 3′ end of the gene cluster is exactly the same, which is also a necessary condition for this study to successfully amplify the complete type 1 pilus gene of APEC by referring to the type 1 pilus gene of UPEC.

FimB and FimE are regulatory proteins of type 1 pili, which control the “switch” of pili. FimB regulates “on” and “off”, while FimE only catalyzes “off” [10]. By comparing the sequences of FimB and FimE of UPEC *E. coli* type 1 pili, the similarities were 98.8% and 99.4%, respectively, with only a few amino acids mutated. Moreover, there was a 51.5% homology between the amino acid sequences of the FimB and FimE proteins. The 25 amino acid sequence (^128^DTRLIQDYLGHRNIRHTVWYTASNA^152^) has only one amino acid difference, W for FimB and R for FimE, with a similarity of 96%. This shows that the expression and regulation of type 1 pili in APEC is the same as that in UPEC.

The FimC chaperone protein is located in the cell periplasm. Although it is not a structural protein of type 1 pili, it is crucial for the assembly of pili. It is responsible for assisting the transfer of pili monomers to the bacterial periplasm to FimD and then assembling the pili. Without the FimC chaperone protein, a subunit complex is formed and pili cannot be assembled [11]. By comparing with UPEC, the similarity of the FimC sequence of NJ05 is 99.52%, with only one amino acid difference. Although the similarity of the FimC sequence of NJ05 with PapD, which is also a chaperone protein, is only 32.9%, there are homology patches in many places. In particular, the 17 amino acids, ^111^LPQDRESLFWMNVKAIP^127^, in the middle of the protein are connected together, with 9 consecutive amino acids being the same and the 5 different ones having the same function. This indicates that different types of pili chaperone proteins perform the same transport mechanism, which needs to be verified by molecular mechanics experiments.

As a propulsion protein, FimD is responsible for exporting the structural protein subunits transported by FimC and assembling them into pili [28]. After comparison, the FimD sequences in NJ05 and UPEC had 87.8% similarity, indicating that, although there were certain amino acid variations, the transport function could still be guaranteed because the variations had not changed the propulsion effect or the presence of the same functional amino acids. The type 1 pili FimD of NJ05 and the mature protein PapC of type P pili, both of which are propulsion proteins, have low homology, but the protein sizes are similar. There are a large number of homology patches between the two, including 15 places with the same arrangement of three consecutive amino acids and 6 places with the same arrangement of four consecutive amino acids. This indicates that there is a certain homology in the amino acid sequences between the propulsion proteins in the assembly process of different types of pili, and that the pili assembly all exports monomers through the same mechanism.

There are many reports on UPEC type 1 pili FimA, which are in a state of continuous mutation [21]. Comparison of FimA-related sequences revealed that the amino acid sequence of the pigeon-derived *E. coli* NJ05 strain and the UPEC type 1 pili FimA subunit had 89~96% similarity, among which the A49088 sequence had the greatest similarity, reaching 96%. The homologous regions are mainly located at the N-terminus, the middle, and the C-terminus. The amino acid sequences in the homologous regions are completely identical, and the variable regions are mainly located at the middle amino acid sequence. This shows that the genes encoding the FimA subunits that make up the pilus rods originated from the same ancestor and are undergoing different degrees of mutation, and the mutation is in a “gradual” state. It is foreseeable that by comparing the amino acid sequences of FimA subunits from more sources, completely identical or more different FimA sequences will appear, and it is even possible to find a transitional type between type 1C pili and type 1 pili. Undoubtedly, this is of great significance for the classification of FimA genotypes and the subsequent screening of type 1 FimA antigens.

It is worth mentioning that the 8th and 14th positions of the mature FimA sequence are both glycine (^8^GTVHFKG^14^), separated by a TVHFK sequence consisting of five amino acids. This is not only the same for UPEC, but also the same for type 1 pili of Shigella and Klebsiella, which are also members of the Enterobacteriaceae family [29]. This shows that this is a key conserved sequence that can ensure that the FimA pilus subunits are inserted from two directions and assembled into pilus rods. This shows that, despite the large variation, the evolutionary pressure has not changed the conservation of key amino acids to ensure the stability of pilus rod formation. This is also seen by comparison with type 1C pili. Compared with the amino acid sequence of the FocA subunit of type 1C pili, FimA has a very low homology of only 67%. However, the amino acids A22, V37, and A93, used to connect the FimA subunits, have not changed, and the amino acids V5, V10, and F12 of the FimA subunit have not changed either. They play a key role in the hydrophobic groove of the previous FimA subunit to insert into the next subunit [24]. The glycine sequences at positions 8 and 14 are also the same, GTVHFKG, which indicates that the common mechanism of pilus rod assembly is still retained during the mutation of type 1 pili to type 1C pili.

The pili rods composed of FimA subunits have a “molecular spring”-like buffering effect. Whether it is UPEC or APEC, in order to slow down the flushing force of human urethra urination or resist the impact of avian respiratory airflow, they can avoid being cleared by the buffering effect of the pili rods assembled by FimA [20]. The results showed that although the FimA subunit of the APEC pili rod had obvious variations compared with UPEC, the key conserved amino acid residues and fragments had not changed, so it was still able to form pili rods, which was very critical for the expression of type 1 pili.

FimH is an adhesion protein located at the tip of the pili. It consists of two parts, the pili binding domain and the lectin binding domain. Its amino acid sequence is very different from that of the type P pili adhesion protein papG [27]. Compared with the type 1C pili FocH of UPEC, the gene sequence and amino acid sequence similarity of the type 1 pili FimH protein of NJ05 are both above 99%, indicating that the FimH protein gene is relatively conserved for APEC and UPEC and follows the same adhesion mechanism in the process of adhering to the mannose receptor. FimH adhesion is closely related to the antigenic epitope, and several amino acids are involved in the formation of the “binding pocket” of the mannose receptor [30]. However, the mutation rate of type 1C pili formed by long-term evolution is very high. In the receptor-binding domain, only 27.6% of the amino acid residues are identical in sequence comparison, especially in the key amino acid variation of the mannose receptor “binding bag”. The amino acids F1, Q133, N135, N138, D140, D141, and F142 of the “binding bag” are mutated, and the three amino acids, Y48, I52, and Y137, that make up the “tyrosine gate” are completely different [31]. This is also the main reason why type 1C pili no longer bind to mannose receptors.

In the pili connecting loop and pili binding domain, the sequence similarity between FimH protein and FocH protein is significantly higher than the proportion of identical amino acid residues in the receptor binding domain. The identical amino acid residues in the connecting loop of FocH are 50%, and only 52.8% of the amino acid residues in the pilus binding domain are the same, and there are also many homology patches. This shows that the pili binding domain of FimH protein of type 1 pili of APEC strains is relatively conserved and is necessary for the connection and assembly of various structural proteins of the pili. In addition, there are a few identical amino acid residues in some regions of FimA, FimF, FimG, and FimH, and it is speculated that they are related to the assembly of pili. These all need to be studied and verified.

In summary, this study constructed an expression vector for the APEC strain type 1 pili gene cluster, which can fully express type 1 pili and has MSHA. At the same time, a *fimH* gene deletion mutant was constructed, which can still express pili but lacks MSHA. The gene cluster DNA sequence was determined, and analysis showed that the gene cluster contained complete *fimB*, *fimE*, *fimA*, *fimC*, *fimD*, and *fimH* genes. Comparison of the amino acid sequence of the encoded protein with the corresponding type 1 pili from UPEC revealed extremely high similarity, with only a high difference in the FimA sequence, but no variation in the reading frame and the amino acids necessary for assembling pili, indicating that the type 1 pili of APEC and UPEC come from the same origin. The comparison also revealed that the chaperone protein FimC and the propeller protein FimD of different pili are similar in size, with many homologous patches and amino acid residues at corresponding positions. There are also a few identical amino acid residues at corresponding positions in some regions of FimA, FimF, FimG, and FimH of the gene cluster itself, and their roles in pili assembly and bacterial adhesion deserve further exploration. This study provides a vector for the biosynthesis of type 1 pili of APEC, which will help to further reveal the pathogenic mechanism of *E. coli* infection in the avian respiratory tract.

## 5. Conclusions

Taken together, recombinant bacteria stable-pUC-*fimBH* and stable-pUC-*fimBG* were constructed; sequencing results showed that the size of the *fimBH* and *fimBG* gene clusters were 8753 bp and 7831 bp, respectively. MHSA indicated that NJ05 and stable-pUC-*fimBH* can agglutinate red blood cells and have MSHA, but stable-pUC-*fimBH* do not have hemagglutination properties and MHSA. TEM results showed that both stable-pUC-*fimBH* and stable-pUC-*fimBG* can express pili. Amino acid sequence analysis indicated that the similarities of FimB of APEC and UPEC was 98.8%, and FimE was 99.4%. The homology between FimB and FimE of NJ05 was 51.5%. The similarities of FimC of APEC and UPEC was 99.52%, and the amino acid homology of FimC of strain NJ05 with PapD of strain 83972 was only 32.9%, but there were many homologous patches between the two proteins. The similarities of FimD of APEC and UPEC was 87.8%, and the amino acid homology of FimD of strain NJ05 with PapC of strain 83972 was low, but there were many homologous patches between them. The similarities of FimA of NJ05 with other APEC strains were 89–96%, and the homology of FimA of APEC and FocA of UPEC was 67%. The gene sequence and amino acid sequence similarities of FimH of NJ05 and UPEC were both above 99%. The amino acid sequence similarity in the pilus binding domain of FimH and FocH was 52.8%, but only 27.6% in the receptor binding domain. The similarity of FimA, FimF, FimG, and FimH of NJ05 with each other was very low, but a few amino acid residues were the same. Further experiments are needed to validate their roles in pili assembly and the pathogenic mechanism of *E. coli* infection in the avian respiratory tract.

## Figures and Tables

**Table 1:** Primers for type 1 pilus gene of *Escherichia coli* in pigeon.

Name		Forward (5′-3′)	Size
Primer 1	*fimBH-F*	**GCTATGACCATGATTACGCC**AAGCTTCCAGCACAGCTAGTGCGCGTCTG	8924 bp
	*fimBH-R*	**GTAAAACGACGGCCAGT**GAATTCTGGCCTACAAAGGGCTAACGTG	
Primer 2	*fimBG-F*	**GCTATGACCATGATTACGCC**AAGCTTCCAGCACAGCTAGTGCGCGTCTG	7927 bp
	*fimBG-R*	**GTAAAACGACGGCCAGT**GAATTCCGGGTTCAGCTGTAGGTATAGGTG	
Primer M13	*M13 rev*	**CAGGAAACAGCTATGAC**	8934 bp
	*M13 fwd*	**TGTAAAACGACGGCCAGT**	

## Data Availability

The original contributions presented in the study are included in the article, further inquiries can be directed to the corresponding author.
